# Nanocellulose size regulates microalgal flocculation and lipid metabolism

**DOI:** 10.1038/srep35684

**Published:** 2016-10-31

**Authors:** Sun Il Yu, Seul Ki Min, Hwa Sung Shin

**Affiliations:** 1 Department of Biological Engineering, Inha University, Incheon, 402-751, Korea

## Abstract

Harvesting of microalgae is a cost-consuming step for biodiesel production. Cellulose has recently been studied as a biocompatible and inexpensive flocculant for harvesting microalgae via surface modifications such as cation-modifications. In this study, we demonstrated that cellulose nanofibrils (CNF) played a role as a microalgal flocculant via its network geometry without cation modification. Sulfur acid-treated tunicate CNF flocculated microalgae, but cellulose nanocrystals (CNC) did not. In addition, desulfurization did not significantly influence the flocculation efficiency of CNF. This mechanism is likely related to encapsulation of microalgae by nanofibrous structure formation, which is derived from nanofibrils entanglement and intra-hydrogen bonding. Moreover, flocculated microalgae were subject to mechanical stress resulting in changes in metabolism induced by calcium ion influx, leading to upregulated lipid synthesis. CNF do not require surface modifications such as cation modified CNC and flocculation is derived from network geometry related to nanocellulose size; accordingly, CNF is one of the least expensive cellulose-based flocculants ever identified. If this flocculant is applied to the biodiesel process, it could decrease the cost of harvest, which is one of the most expensive steps, while increasing lipid production.

With the development of various technologies, people have recently become interested in nano-sized environments and nanomaterials, which have a number of potential applications, including electronics, optics and biology. In biology, nanomaterials exhibit different characteristics and remarkable features when compared with macromaterials, while interacting with cells and tissues. Microalgae offer high potential for diverse nutrients, cosmetics, pharmaceuticals, and exploitation of biodiesel production^1^. In the microalgal biodiesel process, harvesting accounts for 30% of the total cost of the process; thus, its cost-economic process must be developed.

Inorganic compounds, especially metal derived nanoparticles, have been processed and applied to diverse fields, including microalgal harvesting; however, these materials have been found to have many limitations, especially with respect to biocompatibility to humans and the environment^2^. Cellulose constitutes the most abundant renewable polymer, existing in different shapes, sizes and with varying mechanical properties according to its molecular arrangements^3^. Cellulosic nanomaterials are emerging as a class of biomaterials with several desirable properties, including high water absorption capacity, mechanical strength, stiffness and being able to easily conjugate many functional materials with valuable physicochemical properties^4^. Because of these properties, these materials have many potential applications in electronics, food and tissue engineering. In the microalgae field, cation-modified cellulose nanocrystal (CNC) has recently been applied to induce flocculation for dewatering during biofuel production^5^. The results revealed that the cation-modified CNC flocculated microalgae via ion neutralization, but anion-modified CNC did not. However, cation modification weights the environmental and harvesting process costs; thus, if the original form of anionic cellulose nanomaterials derived by acidic hydrolysis could flocculate microalgae, they would be a good candidate for microalgal harvesting.

In this study, we investigated the size effects of cellulose nanomaterials on microalgae flocculation and lipid metabolism. Cellulose nanofibrils (CNF) flocculated microalgae, despite having an anion surface, and lipid synthesis was highly upregulated via a mechanotransduction-mediated mechanism. CNF induced flocculation via a mechanical interaction based on geometric properties such as its nanocellulose size and hydrogen bonding (Fig. 1). Based on these findings, CNF is a candidate flocculant that is environmentally-friendly and inexpensive.

## Results

### Size-dependent separation of CNC and CNF

**Figure 2:**
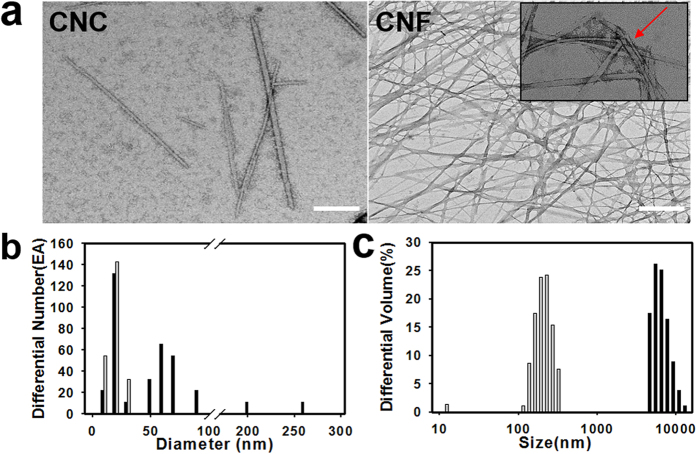
Morphology and size distribution of tunicate-derived CNC and CNF. (**a**) TEM images of CNC (scale bar 100 nm) and CNF (scale bar 200 nm). Red arrow indicated amorphous region of CNF. (**b**) Diameter distribution of CNF fibrils. (**c**) Size distribution of CNF fibrils. Distributions of diameter and size of CNF (black bar) were compared with those of CNC (gray bar).

To confirm whether the hydrolyzed tunicates were separated into CNC and CNF according to their size, TEM and ELS analyses were conducted. As shown in Fig. 2a,b, hydrolysis of tunicate cellulose revealed crystalline nanocellulose. CNC expressed rod-like morphology with an average width of 19.04 nm. Conversely, CNF showed a flexible morphology with a width of 54.24 nm. CNF showed many networks formed by entangled flexible nanofibers and crystalline. Size distributions of CNC and CNF were also quantified using ELS (Fig. 2c). CNC and CNF showed 201.49 nm and 6.85 μm in average size, respectively. These results show that CNC and CNF were well separated according to size. Both CNC and CNF were negatively charged, following zeta potential results (Supplementary Fig. 1).

### CNF flocculates microalgae

**Figure 3:**
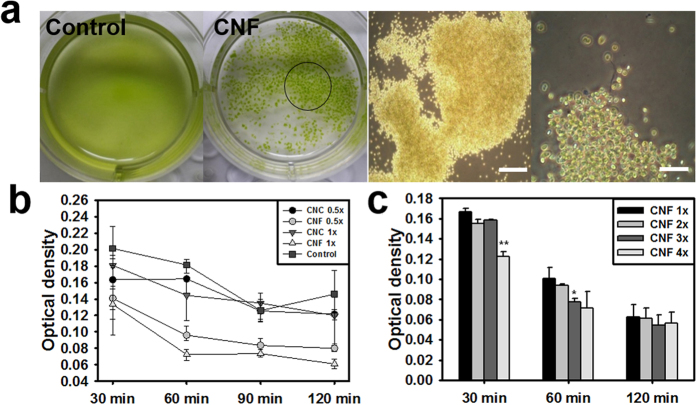
The effect of CNF on microalgal flocculation. (**a**) Microscopic observations of CNF flocculation of microalgae (black circle, scale bar: left 200 μm, right 50 μm). (**b**) Optical density of supernatants after microalgal flocculation of CNF. Control is microalgae without treatment of CNC or CNF. Supernatants in CNF media showed low optical density since CNF flocculated microalgae (CNF and CNC 1× concentration : 0.47 mg/ml). (**c**) The effect of CNF concentration on microalgal flocculation (*p < 0.05, **p < 0.01, ***p < 0.001).

Flocculation was the typical interaction between CNF and microalgae. When poured into microalgae culture, CNF aggregated the microalgae, but CNC did not (Fig. 3a). The Optical density of the supernatant after flocculation will decrease in the presence of fewer microalgae. As expected, the OD of supernatant for all CNF concentrations decreased with time, implying that flocculation progressed gradually with time (Fig. 3b). Moreover, microalgae flocculation increased as the CNF concentration increased (Fig. 3b,c). Flocculation speed was accelerated as CNF concentration increased but flocculation level was saturated to one point, regardless of CNF concentration (Fig. 3c).

### Ions and sulfuric moieties do not affect microalgal flocculation of CNF

**Figure 4:**
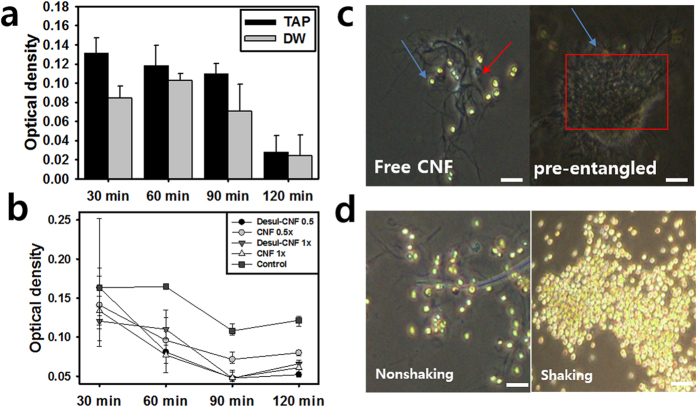
Chemical or mechanical effects of CNF on microalgal flocculation. (**a**) Ion effect of media on microalgal flocculation of CNF. DW without ions flocculate microalgae faster than ion-containing TAP. (**b**) The effect of sulfuric moiety of CNF on microalgal flocculation. Control is microalgae without CNF treatment. No significant difference was observed between sulfured and de-sulfured CNF. (**c**) Freely moving CNF (red arrows) flocculate microalgae (blue arrows) better than pre-entangled CNF (red box). Scale bar 50 μm. (**d**) Hydrodynamic force accelerates microalgal flocculation of CNF (left: no hydrodynamic force, right: Hydrodynamic force, scale bar 50 μm) (*p < 0.05, **p < 0.01, ***p < 0.001).

To investigate whether the flocculation mechanism is dependent on ionic strength of culture media, microalgal flocculation with CNF was compared between TAP growth media and DW. The flocculation rate was faster in DW for all CNF concentrations, but no significant difference was observed in flocculation at the final time (Fig. 4a). To determine the effects of sulfuric moieties on flocculation, desulfated CNF was also examined. Desulfated CNF showed reduced sulfur contents with respect to reaction time, with 0% at 6 hr, while unmodified CNF was found to consist of 44.61% carbon, 5.96% hydrogen, and 0.24% sulfur contents (Table 1). Microalgal supernatants after mixing with unmodified and desulfated CNF showed similar OD values of 0.059 and 0.051, respectively, indicating that the sulfuric moiety does not affect algal flocculation (Fig. 4b).

### Nanofibrous network of CNF is relevant to microalgae flocculation

While investigating the structural effects of CNF on microalgae flocculation, we found that free-moving CNF fibrils in static solution flocculated microalgae better than the pre-entangled CNF network (Fig. 4c). Moreover, the OD of CNF supernatant without cells decreased faster in DW than in TAP growth media over 30 min, even though both reached a similar saturation level at the final time and no significant difference was measured (Supplementary Fig. 2). This is in consistent with the experimental results that CNF flocculated microalgae faster in DW than in TAP. Taken together, these results imply that the CNF network formation in static solution would be relevant to microalgae flocculation. Fluid momentum flux influences mobility and network formation of CNF nanofibrils, causing variations in microalgal flocculation. To confirm this, agitation was applied to CNF and variations in microalgal flocculation were observed. As shown in Fig. 4d, agitated CNF showed better flocculation than static CNF.

### CNF nanofibrous network was formed by amorphous structure and hydrogen bonding

**Figure 5:**
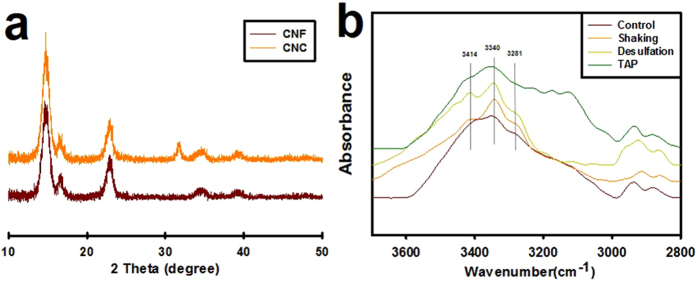
Investigation of crystallinity and hydrogen bonding of CNF using XRD or FT-IR. (**a**) X-ray diffraction of CNF and CNC. In order to compare crystallinity of CNF with that of CNC, crystallinity index(CI) value was calculated as follows (CI = I_crystal_/(I_crystal_ + I_amorphous_) × 100). (**b**) FT-IR spectra of CNF. Control is spectra of CNF in DW. 3281 cm^−1^ and 3340 cm^−1^ indicates intra-hydrogen bonding of CNF, and 3414 cm^−1^ means inter-hydrogen bonding of CNF.

Crystallinity levels were determined for CNF and CNC (Fig. 5a) using XRD. After applying Eq. 1, the crystallinity index (CI) values of CNF and CNC were found to be 92.59% and 96.18%, respectively, which implies that CNF has an amorphous structure relative to CNC. Hydrogen bonding is relevant to inter- or intra-cellulose interactions (30). FT-IR absorption spectra were analyzed for CNF with respect to culture media, sulfur moieties, and fluid dynamics (Fig. 5b). First, CNFs in DW and TAP media were compared by cellulose IR of OH bonding (hydrogen stretching region: 3800–2600 cm^−1^), of which hydrogen bonding were indicated 3414 and 3340 cm^−1^ intramolecular, and 3281 cm^−1^ intermolecular bond. Both had no significant difference in intramolecular hydrogen bondings but CNF in DW displayed more distinct peak than in TAP, implying that CNFs in DW interconnected themselves via OH bonding. Evaluation of the effects of sulfur contents revealed that desulfated CNF displayed 3340 cm^−1^ and 3414 cm^−1^ peaks that were sharper than the non-modified one, implying that desulfated CNF undergoes stronger hydrogen bonding in that region. Finally, agitated CNF presented sharper peaks than static ones at 3340 cm^−1^ and 3414 cm^−1^ peaks. These findings indicate that agitation induced strong hydrogen bonding of intra-CNF bonds.

### Analysis of intracellular lipid contents using nile red staining and GC-MS

**Figure 6:**
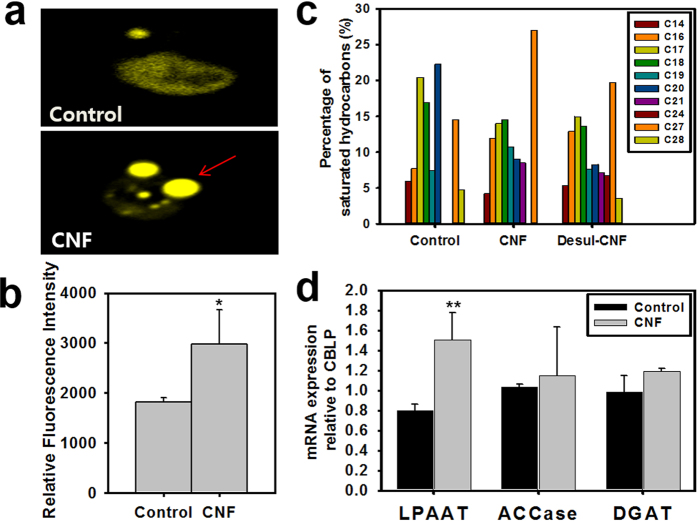
Upregulation of lipid synthesis of microalgae on CNF. (**a**) Confocal microscopic images of nile red stained lipid droplets (red arrows) of *C. reinhardtii*. (**b**) Lipid quantification of *C. reinhardtii* by spectrofluorometer. (**c**) GC-MS analysis of free fatty acid of *C. reinhardtii* on CNF. (**d**) Expressions of mRNAs related with lipid synthesis of CNF flocculated microalgae. mRNA expression levels were normalized with housekeeping gene (CBLP) (*p<0.05, **p<0.01, ***p<0.001).

Lipid droplets were examined on microalgae in CNF by nile red staining. Microalgae cultured on CNF showed increased lipid bodies relative to control (Fig. 6a), and this difference became distinct as culture time increased (Supplementary Fig. 4). Following quantification of lipid bodies using a spectrofluorometer, total lipid contents of microalgae cultured on CNF increased by two times relative to that of suspension culture observed on day 1 and 3, but was slightly lower on day 5. In addition, total lipid content was higher in CNF relative to in suspension (Fig. 6c). Hydrocarbon contents of microalgae was measured using GC-MS. Microalgae flocculated on CNF was examined to have lipids of long carbon chain (above 21 carbon contents) more than free microalgae (Fig. 6b). Conversely, most lipids with short carbon chain decreased. These phenomena also appeared for the desulfated CNF.

### Identification of mRNA expression levels for lipid synthesis

To identify why lipid synthesis was upregulated in CNF environment in a transcript level, mRNAs of ACCase, DGAT, LPAAT, which are associated with fatty acid synthesis, were quantified. In Fig. 6d, the three mRNAs of CNF-flocculated microalgae increased compared with those of free microalgae. Especially, LPAAT that catalyze to phosphatidic acid was increased about two-fold.

### Mechanotransduction of CNF to microalgae

**Figure 7:**
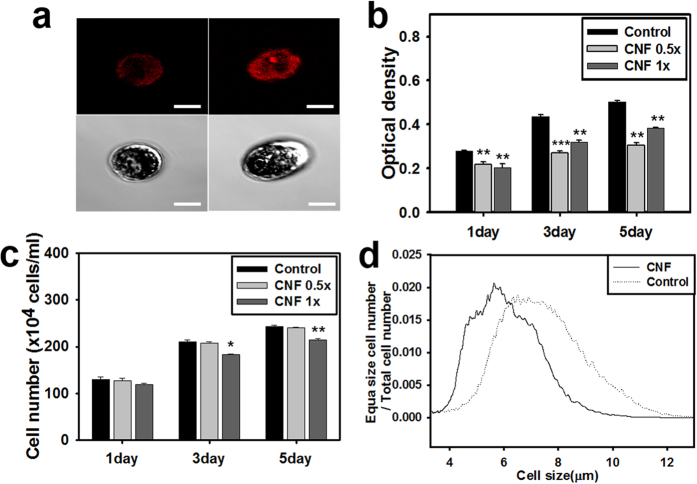
Calcium influx, cell mass and number, and size distribution of CNF flocculated microalgae. (**a**) Fura-2 stained calcium ions of microalgae without CNF (left) and without CNF (right). Scale bar 10 μm. (**b**) Measurement of total cell mass of microalgae by spectrophotometer (680 nm). (**c**) Measurement of cell number of microalgae using hemocytometer. (**d**) Cell size measurement of microalgae using coulter counter (*p<0.05, **p<0.01, ***p<0.001).

To evaluate the mechanotransduction of microalgae cultured on CNF, intracellular calcium contents were measured by Fura-2 staining. Microalgae in CNF have stronger fluorescent intensity than in suspension (Fig. 7a). Chlorophyll contents of microalgae in CNF decreased about 30% (Fig. 7b), which indirectly indicates that cell mass decreased under CNF flocculation. The cell number of microalgae in CNF is also in accordance with the chlorophyll trend, with decreases being observed in CNF relative to that in suspension and greater decreases being observed with time (Fig. 7c). Cell size also decreased by 1 μm in CNF relative to in suspension (Fig. 7d).

## Discussion

Microalgal harvesting has high costs that must be overcome before it can be effectively applied for biodiesel production. This study was conducted to develop an unmodified CNF to both lower harvesting cost and increase lipid production, utilizing the size effect of CNF on its geometric structure and microalgal mechanotransduction. Conventional sulfuric acid treatment is a cost-effective way to yield cellulous nanomaterials and thus adequate to obtain cellulose nanomaterials, even though the reaction could be affected by acid concentration, acid type, reaction time, and temperature^6^. Tunicate was selected for this study since cellulose nanomaterial with a wide range of sizes can be obtained relative to other sources^7^. As shown in Fig. 2, the sizes ranged from 200 nm to 50 μm. Moreover, elemental analysis identified the existence of sulfur moieties (Supplementary Fig. 3), which were generated by sulfur esterification modifications^8^. Following size separation, the resulting long CNF and short CNC were assessed for the interactions with microalgae, respectively.

CNF unexpectedly flocculated microalgae, which, to the best of our knowledge, has not previously been reported (Fig. 3). Anion CNC expressed rare flocculation, which is coincident with the results of previous studies^9^. In addition, microcrystalline cellulose did not cause microalgal flocculation^10^. Taken together, these findings indicate that CNF flocculant is unique and size-dependent. There are several possible explanations for the flocculation mechanism. Electrostatic force might have anionic CNF flocculate negative microalgae with the help of inner-connecting cationic metal ions. This ion bridge flocculation often occurred in ionic flocculants, and tunicate derived cellulose crystalline structures may undergo this process because of their strong zeta potential. However, as shown in Fig. 4a, the deionized water (DW) that removed metal ions caused faster flocculation of microalgae. This implies that the ion bridge is not a major mechanism for flocculation. Conversely, the slower flocculation in TAP might be caused by flocculant inhibitors, as previously reported^11^. To determine if the sulfuric moieties could affect flocculation even though ion bridges were determined not to be a major mechanism, the effects of de-sulfured CNF on microalgae flocculation were investigated. However, de-sulfation did not induce significant changes in flocculation (Fig. 4b). These findings indicate that flocculation is not caused by electrostatic interactions, but rather by other properties of CNF.

Another possible mechanism is formation of a CNF network, followed by mechanical entrapment of microalgae. CNF was shown to be entangled by its flexible nanofibrils (Fig. 4c, Supplementary Fig. 2). Similar phenomena have previously been discussed in terms of shear-lag theory and intra- and inter-forces such as hydrogen, hydrophobic, and Van der Waals forces^3^,^12^. These intra-CNF interactions were thought to result in a nanofibrous network that entrapped freely moving microalgae because free-moving CNF fibrils flocculated cells better than pre-networked CNF fibrils (Fig. 4c). These findings also show that nanocellulose network geometry is very sensitive to microalgae flocculation. The suggested flocculation mechanism is supported by the fact that hydrodynamic momentum flux accelerated the flocculation rate (Fig. 4d). If freely moving microalgae were mechanically entrapped into the network, hydrodynamic flux would increase the collision frequency between microalgae and the nanocellulose network. Conclusively, attractive forces and mechanical entanglement caused CNF to form a nanofibrous mat, which was followed by microalgae flocculation via mechanical entrapment.

Generation of the CNF nanofibrous network via mechanical entanglement and attractive forces was evaluated in terms of amorphous structure and hydrogen bonding. In view of mechanical entanglement, the longer nanofibrils, which have more flexible morphology, lead to mechanical entanglement among nanofibrils more easily according to the shear-lag theory^13^,^14^. Based on the CI values of CNF (Fig. 5a), the flexibility of CNF increased due to the amorphous region. If CNF consists of rigid beta-sheet like CNC, then it will be less flexible, despite its long length, and would be stacked to maximize the contact area, resulting in its not being entangled. In view of attractive forces, hydrogen bonds are the strongest intermolecular interactions except for electrostatic force, and they can induce flocculation or form structures among freely moving nanomaterials^15^,^16^. In this sense, we could assert that ions in TAP media played a role in inhibition of hydrogen bonding between CNF, resulting in CNF in distilled water being flocculated faster than in TAP media. Strong free ions in TAP media could inhibit attraction among partial polar groups of inter hydrogen bonds of CNF, resulting in slower CNF intra-network formation. Comparison of FT-IR data between CNF in DW and in TAP media revealed that some hydrogen bond peaks were covered and broadened by ions in the TAP media, which would influence hydrogen bonds among CNF. Accordingly, early flocculation inhibition by TAP media might have occurred via this effect. In addition, comparison of normal and desulfated CNF revealed that, although there was sharper hydrogen bonding in a given region, no significant differences were detected in hydrogen bonds. These findings imply that any intra-fibril interactions were not significantly changed by desulfation of CNF. This is coincident with the fact that both CNFs had similar flocculation speeds. However, agitated CNF showed sharper peaks at 3340 cm^−1^ to 3414 cm^−1^ than static CNF. This phenomenon demonstrated that agitation induces a tight network among CNF. This characteristic is related to the compact and faster network formation due to mechanical stress on CNF solution^14^,^17^,^18^.

The mechanically entrapped microalgae naturally modify their physiology, adapting themselves to the flocculated environment. Mechanical stress in microalgae is transmitted through the mechanosensitive ion channel of calcium ion^19^. As seen in Fig. 7, following calcium influx in response to mechanical stress, microalgae compensated to reduce cell growth and size to protect themselves. In this respect, CNF are believed to impart mechanical stress to the entrapped microalgae, resulting in lower cell growth and small cell size. Mechanotransduction via calcium influx also upregulates lipid contents. To determine if this mechanism is applicable to CNF, intracellular lipid contents of microalgae were measured. As shown in Fig. 6, when compared with the control, CNF were shown to increase the intracellular lipid contents of microalgae.

In addition, GC-MS data showed that hydrocarbon of microalgae was also converted to long carbon chain. This upregulation and conversion of lipids could be the result of compensation for the downregulation of cell growth and size^20^. Conclusively, the flocculation exerts mechanical stress on microalgae, invoking calcium influx through mechanosensitive ion channels, resulting in decreased cell growth and size and upregulated lipid synthesis.

Biocompatible anionic CNF was studied for microalgae harvesting and high lipid production. CNF was transformed to a nanofibrous network by attractive forces such as hydrogen bonding and mechanical entanglement, followed by microalgal entrapment. This study is the first to report that anionic CNF could be a flocculant. In addition, flocculated microalgae excited mechanotransduction relevant to calcium ion influx, which drove metabolic flux to up-regulate syntheses of lipids and hydrocarbons, a self-defense pathway, at the expense of cell growth. Therefore, CNF is a biocompatible and low-cost flocculant that makes it easier to harvest microalgae while increasing lipid and hydrocarbon contents. Accordingly, this nanomaterial has the potential to dramatically decrease the economic cost of the microalgal biodiesel process.

## Methods

### Preparation of tunicate CNC and CNF

The integuments of tunicates were separated and dried in an oven, which were then immersed in 5% potassium hydroxide (Daejung Chemistry, ROK) aqueous solution at 80 °C for 3 days. Next, aqueous solutions of 0.17% acetic acid (Daejung Chemistry, ROK) and 0.34% sodium hypochlorite (Daejung Chemistry, Republic of Korea, 4% < chlorine) were applied to the integuments at 60 °C and the solutions were exchanged hourly until the tunicate’s pigments were removed, after which they were washed with distilled water. Samples were then filtered with a 1 mm size sieve, after which the powered integuments were treated with 60% (w/v) sulfuric acid aqueous solution (Daejung Chemistry, ROK) and stirred for 20 min at 60 °C. Following the reaction, the temperature was reduced to 0 °C to stop the hydrolysis reaction. Residual sulfuric acid was subsequently neutralized with distilled water until pH 6, after which the samples were sonicated for 30 min Crude hydrolyzed cellulose was filtered using 0.7 μm whatman microfiber filters (GE, United Kingdom) under vacuum to separate CNF and CNC. For desulfurizing the integuments, 2N NaOH solution was added and reacted for 6 hr at 60 °C and subsequently dialyzed for 7 days to remove NaOH and remaining metal ions.

### Characterization of CNC and CNF

The morphology and size of CNC and CNF were observed by transmission electron microscopy (CN200, Philips, Netherland). Briefly, CNC or CNF were collected from 1 ml suspensions, then dyed with 1% uranyl acetate for 5 min in a dark room. After sequentially washing 3 times with DW, removing bubbles on the top of the grid with a pipette, and drying the TEM grid using a vacuum, TEM images were taken. Image J software (National Institutes of Health, USA) was used to calculate the size and width of CNC or CNF on TEM images.

Surface charge and particle size of CNC or CNF was measured using zeta potential and dynamic light scattering (ELS-Z, Otsuka, Japan). The crystallinity index of samples was examined by x-ray diffraction (XRD) (X’Pert PRO MRD, Phillips) at 40 kV and 30 mA tube current at 2θ angles from 10° to 90°. Sulfuric contents were measured by elemental analysis (Tecan, Swiss).

Fourier transform infrared spectrometer (VERTEX 80 V, Bruker, USA) was used to investigate the interaction of specific inter and intra cellular hydrogen bonding. Aggregated, desulfated and different solvent (TAP media) of CNF solution(1 mg) was embedded into KBR pellet and dried water in a oven during 12 hr and detected with OPUS 6.0 software(Bruker, USA) in 4000 ~ 400 cm^−1^ range.

### Microalgae culture and flocculation assays

*Chlamydomonas reinhardtii* strain cc-124 was cultured in a shaking incubator (80 rpm) at 23 °C under a 12 hr light and 12 hr dark cycle. TAP media (Gibco) was used as a basal media. Cell mass was estimated based on the optical density at 680 nm using a spectrophotometer (Tecan, Swiss). To identify aggregation of microalgae with CNC or CNF, various volumes of CNC or CNF were then added to the C. reinhardtii culture (optical density = 0.45 at 680 nm) to adjust it to a final cell culture volume of 10 ml. Sedimentation level was measured at 680 nm using a spectrophotometer.

### Measurement of optical density, cell number and size, and chlorophyll contents

Cell viability was determined based on the cell number and chlorophyll contents every 24 hr for 3 days. Various concentrations of CNC and CNF were added into the culturing microalgae, after which each sample was collected and amended with 2.5% glutaraldehyde (Samchun, Korea) in PBS, separated by centrifugation to remove the TAP media and glutaraldehyde. Total cell mass was confirmed using a spectrophotometer at 680 nm. In addition, cell number was determined by direct cell counting using a hemocytometer (Marienfeld-Superior, Germany), while cell size was measured using coulter counter (Beckman Z series Coulter counter, USA). Cell mass of microalgae were measured using spectrophotometer (Tecan, Swiss). Briefly, cell pellets were treated with 100% methanol for 24 hr at 4 °C. Optical density was measured at 680 nm to measure cell mass contents.

### Analysis of intracellular lipid contents

Nile red staining was applied to monitor intracellular lipid contents of microalgae. First, microalgae cultured with CNF were fixed with 2.5% glutaraldehyde in PBS. After discarding the sample’s media solution using a centrifuge (12,000 g, 1 min), the cell pellet was resuspended with PBS, then mixed with Nile red dye (Sigma Aldrich, 2 μg/ml in acetone) at a 1:3 volume ratio (dye:sample) and finally incubated for 10 min at 40 °C in the dark. Intracellular lipid images were obtained by confocal microscopy (Carl Zeiss LSM510 meta, Germany) with excitation 543 nm and emission 630 nm wavelengths. Quantified intracellular lipid contents were confirmed using a spectrofluorometer (Hitachi F-4500, Japan). The same Nile red stained sample was used to measure the quantity of lipid contents under the following conditions: excitation slit 10 nm, emission slit 10 nm, photomultiplier 400 V, excitation wavelength 480 nm, emission filter wavelength 570–590 nm). Hydrocarbon contents of microalgae was examined using 6890N GC system (Agilent Tech, USA). Details to process of GC operation was indicated below. J&W GC column DB-5 ms (Agilent Tech, 30 × 0.25 × 0.25, USA), Inlet temp 280 °C, Injection volume 1 μl, Oven temperature 30 °C to 300 °C at 10 °C/min. Mass spectrometry was measured by Pegasus III (Leco, USA).

### Measurement of Calcium Influx by Fura-2

The intracellular calcium concentration was detected by Fura-2 staining (Sigma Aldrich). Microalgae samples cultured with CNF were centrifuged at 13,500 g for 1 min, after which the media was exchanged with NMG^+^/K^+^ buffer (1 mM KCl, 200 μM K^+^ BAPTA, adjusted with N-methyl-D-glucamine, 5 mM HEPES, and 10 mM HCl to pH 5.6 using 1 mM sulfinpyrazone) and 3 μM Fura-2 dissolved in DMSO. The sample was then resuspended in a 6 well plate, incubated for 24 hr at 23 °C and collected. An image of the calcium influx of microalgae was taken by confocal microscopy at an excitation of 380 nm and emission of 510 nm.

### Analysis of mRNA using Real-time qRT-PCR

Microalgal mRNA was collected using Trizol reagent (Takara, Japan). The extraction was conducted according to the manufacturer’s guidelines. Total mRNA preservation was checked by agarose gel electrophoresis using safe shine green (Biosesang, Republic of Korea). After synthesizing cDNA using a QuantiTect Reverse Transcription Kit (Qiagen, Germany). Real Time qRT-PCR was conducted according to the manufacturer’s instructions. Briefly, the specific volume ratio of the cDNA tablet, 2× SYBR in a QuantiFast SYBR Green PCR kit and primer were mixed and diluted to 20 μl. Data describing specific cDNA were expressed in the form of delta-delta Ct (2^(−∆∆2γ)^) and quantified using the Rotor-Gene Q Series Software (Qiagen, Germany). The 2^(−∆∆2γ)^ value was normalized against the expression level of CBLP, a housekeeping gene.

### Statistical analysis

All experiments were conducted in triplicate and the results are presented as the means ± SD. And Using student’s t-test, significant difference between groups was identified at p-value (*<0.05, **<0.01, ***<0.001) to represent difference.

## Additional Information

**How to cite this article**: Yu, S. I. *et al*. Nanocellulose size regulates microalgal flocculation and lipid metabolism. *Sci. Rep.* **6**, 35684; doi: 10.1038/srep35684 (2016).

**Publisher’s note:** Springer Nature remains neutral with regard to jurisdictional claims in published maps and institutional affiliations.

## Supplementary Material

Supplementary Information

## Figures and Tables

**Figure 1:**
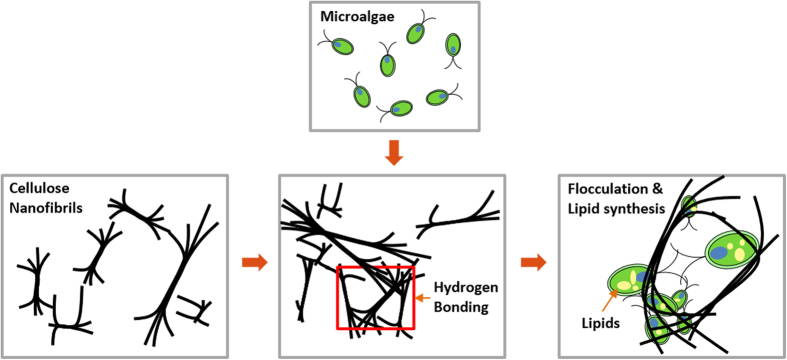
Schematic design of CNF induced microalgal flocculation and lipid synthesis. Freely moving CNF form network by their entanglement or hydrogen bonding, after which the CNF network flocculates freely moving microalgae and upregulates lipid synthesis.
