# Supplementary material for: Nanocellulose size regulates microalgal flocculation and lipid metabolism
Source: Sci Rep. 2016 Oct 31;6:35684. doi: 10.1038/srep35684 (PMC5086845; doi:10.1038/srep35684)
Supplement: Supplementary Information [file srep35684-s1.pdf]

Nanocellulose size regulates microalgal flocculation and lipid metabolism

Sun Il Yu[1], Hwa Sung Shin[1, *]

[1]Department of Biological Engineering, Inha University, Incheon, 402-751, Korea

*Corresponding author.

Hwa Sung Shin, hsshin@inha.ac.kr, Tel: 82-32-860-9221, Fax: 82-32-872-4046

This file includes:

3 figure which are supporting our research.

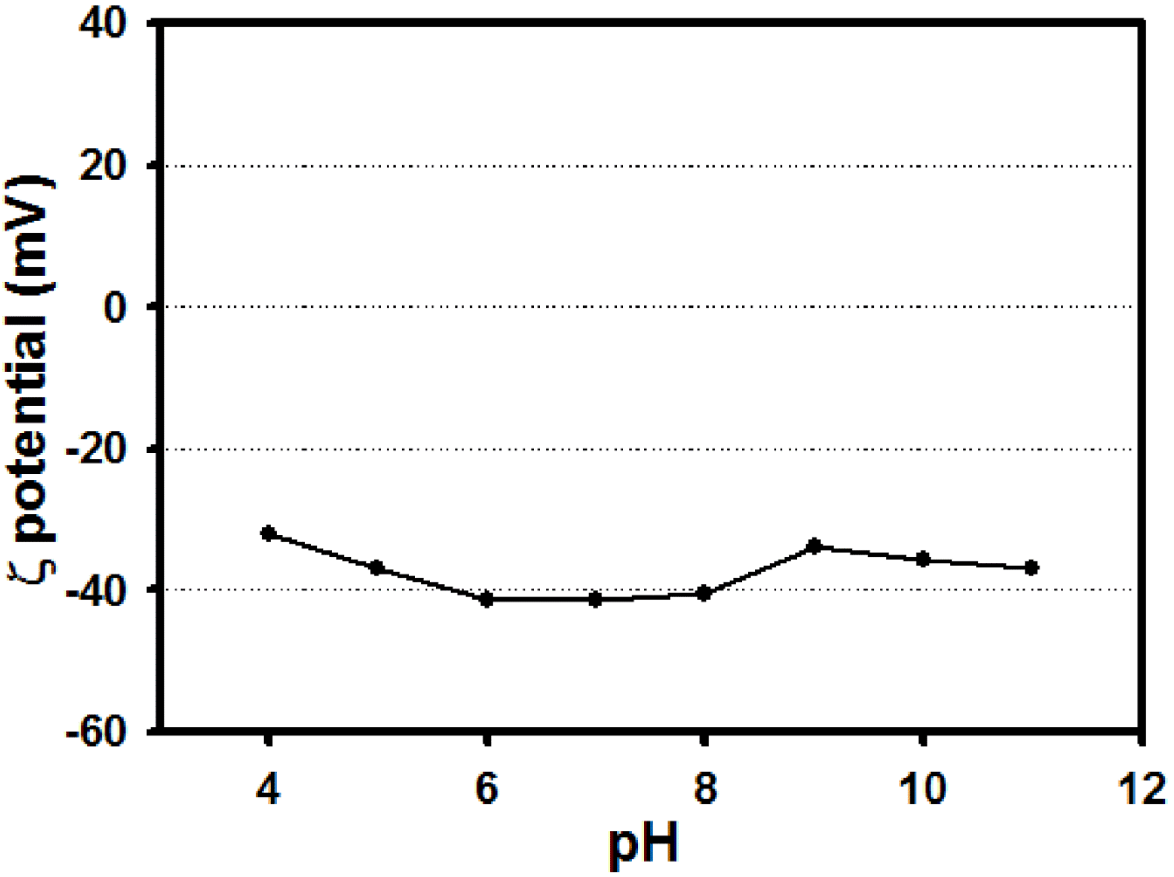

Supplementary Figure 1. Zeta potential of CNF in DW.

CNF has negative charge under conditions where microalgae can be cultivated (from pH 4 through pH 11)

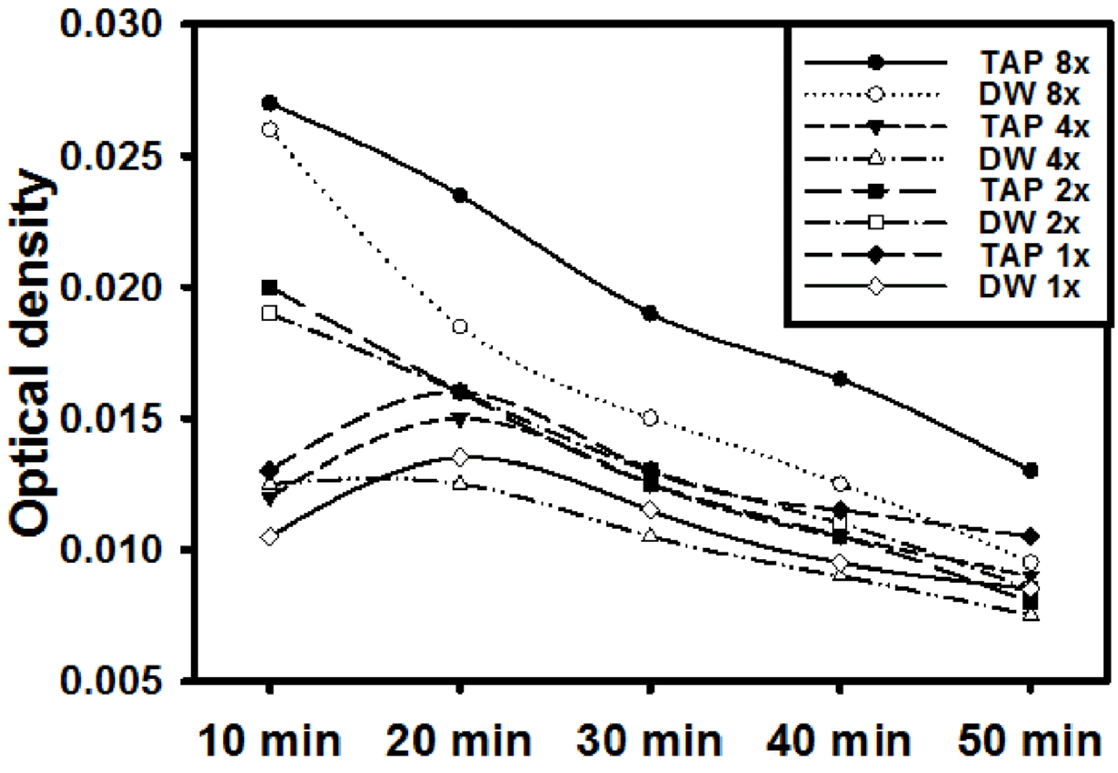

Supplementary Figure 2. CNF aggregation in TAP or DW with respect to time.

Optical density measurement of supernatants after CNF aggregation at 400 nm using spectrophotometer. CNF in DW aggregated faster than those in TAP. After CNF aggregation, optical density decreases. CNF solution displayed the maximum absorbance at 400 nm wavelength.

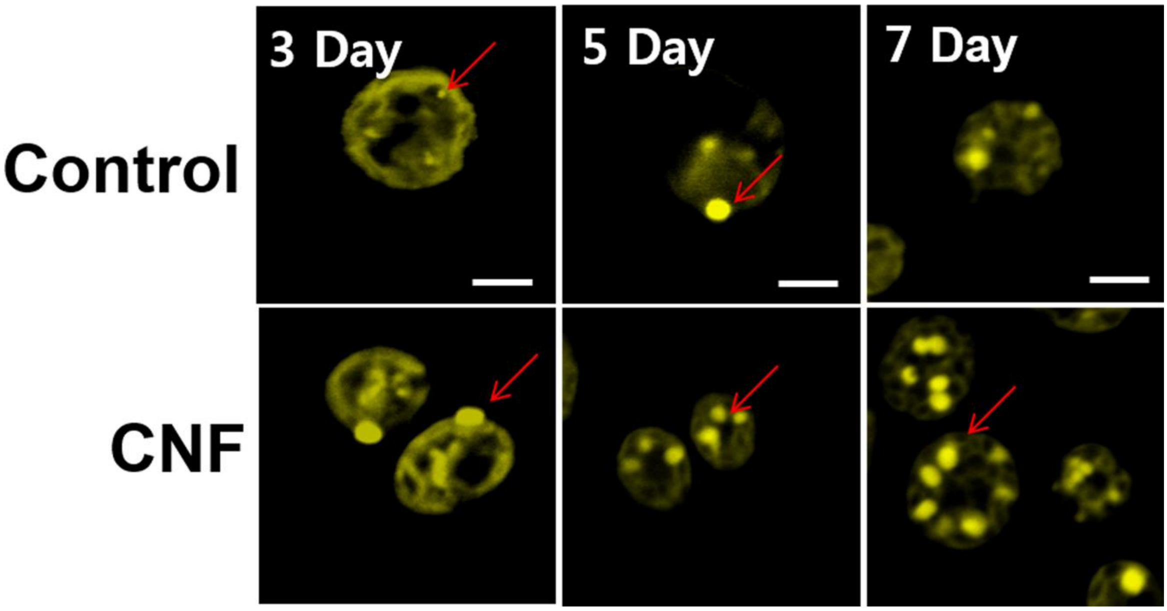

Supplementary Figure 3. Comparison of lipid droplets synthesized in microalgae cultured in CNF with those of free microalgae.

Confocal microscopic images of nile red stained lipid droplets. Red arrows indicates algal lipid droplets. (scale bar : 10 μm)
